# Detection and quantification analysis of chemical migrants in plastic food contact products

**DOI:** 10.1371/journal.pone.0208467

**Published:** 2018-12-05

**Authors:** Shasha Qian, Hanxu Ji, XiaoXiao Wu, Ning Li, Yang Yang, Jiangtao Bu, Xiaoming Zhang, Ling Qiao, Henglin Yu, Ning Xu, Chi Zhang

**Affiliations:** 1 Nanjing Institute of Supervision & Testing on Product Quality, National Supervision & Testing Centre for Food & Food Addictives, Jiangsu Center of Supervision & Testing on Green Degradable Material Quality, Nanjing, PR China; 2 Southeast University, State Key Laboratory of Bioelectronics, Nanjing, PR China; Agricultural University of Athens, GREECE

## Abstract

Plastic food contact materials (FCM)-based products were widely used in everyday life. These products were normally imposed to strict regulations in order to pass the enforcement tests of compliance as a prefix condition. However, even in these “qualified” materials, unknown chemical substances, not involving in legislation lists, could migrate from FCM. In this perspective, the present work aims to thoroughly analyze by means of Gas Chromatography-Mass Spectrometry (GC-MS) the different substances/migrants in 120 qualified FCM plastic products. Unexpectedly, among the identified compounds (nearly 100), only 13% was included in the permitted list of Commission Regulation EU No 10/2011. All the identified compounds were classified into 11 categories according to their chemical structure and the FCM type, whereas toxicology data were in addition analyzed. Each plastic type exhibited different preferences of chemical migrants. Fortunately, most of the compounds identified were of low toxicity, and only 4 chemicals were included in priority lists and previous literature reports as potential risk factors. Subsequently, the accurate amount of these 4 chemicals was determined. The amount of Bis(2-ethylhexyl) adipate (DEHA) and Bis(2-ethylhexyl) phthalate (DEHP) were lower than the SML in Commission Regulation EU No 10/2011, and that of stearamide was under the recommended use quantity. The 2,4-di-tert-butylphenol (2,4-DTBP) was widely exist in the investigated FCM products. Among them, the highest level is obtained in polypropylene/low density polyethylene (BOPP/LDPE) materials, up to 45.568±31.513 mg/kg. In summary, a panel of unlisted chemical migrants were discovered and identified by GS-MS screening. The results implied that plastic FCMs were not so “inert” as they usually considered, and further safety evaluation should be performed toward the complete identification of new substances in FCM products.

## Introduction

In general, FCMs (food contact materials) are intended to protect food products from contamination, thus extending their shelf life, while facilitating their transportation and storage. In recent decade, particular attention has been focused on food safety issues that may be related to FCMs. For instance, two major issues have concerned the food industry in Europe: BPA (bisphenol A) in Polycarbonates and benzophenone in Printed cardboard[1].Chemicals with molecules and ions of small size (below 1000 Da) that can migrate from packaging into food are considered as one of the most important risk factors in food supplying chain. The nature and the amount of these chemical migrants are strongly depending on the packaging material, contacted food and migration conditions, etc[2–5].Among these chemicals, the typical contaminants of high risk have been summarized and evaluated[6, 7].

Polymeric materials are the most extensively employed FCM in food packaging, due to their wide applicability and low price. Many traditional polymers, such as poly vinyl chloride (PVC), polyvinyl acetate (PVA), polyethylene (PE), polypropylenes (PP) and polycarbonates (PC), constitute the main components of FCM plastics[8]. In addition to the basic polymers, various additives are usually employed in order to obtain the desired properties and/or simplify the manufacturing process. Fillers and softeners (plasticizers) might be added in high concentrations to increase volume and/or weight and improve softening, respectively[9–11]. Other additives, such as heat and light stabilizers, antimicrobials, antioxidants, colorants, UV absorbers, light screening pigments and dehydrating agents, are also used in relatively small amount[12–14]. These additives, along with the impurities of starting materials and the contaminants from material recycling, may all remain in the final products exhibiting high mobility. These components are the main source of hazard migrants with potential health risks in amount beyond the toxicological threshold.

In order to ensure the quality and safety of food contacted plastics, most nations have established lists of “allowed” and “non-allowed” substances along with their limits. Based on the toxicology data and risk assessment, European Regulation EU No 10/2011 specifies the allowed substances in plastic food package along with the specific migration limits (SML)[13, 15]. The equivalent law to EU No 10/2011 in China is GB9685[16].

In general, the enforcement test of compliance with the legislation includes two separate experiments: overall migration is initially evaluated using food stimulants to mimic realistic migration; meanwhile, according to different plastic type, the levels of specific migrants, also known as hazard factors, are determined in the stimulants. The results are compared with the overall migration limit (OML) and SML to confirm whether the quality of the products is in accordance to specifications[17, 18].

Plastic FCMs may contain thousands of different molecules, in which more than 300 have been considered as risk factors[19, 20]. Both in EU No 10/2011 and GB9685 directives, hundreds of substances are specified with SML, whereas only less than 40 substances have standard validated methods to assess their specific migration[21]. Numerous researches have established various analytical methods to determine the different migrants in plastic materials and food stimulants. Gas chromatography (GC) and Gas Chromatography-Mass Spectrometry (GC-MS) is the main techniques that can be employed in practice[22, 23]. Besides, Gallart-Ayala et al.[24] summarized the recent advances in relation to the analysis of food-packaging contaminants by Liquid Chromatography-Mass Spectrometry (LC-MS).

Although reliable methods for the determination of certain specific migrants are widely established, there is no study, to the best of our knowledge, on overall migrants’ pattern in FCM by following a scanning approach. The latter is of particular importance, taking into account that in a plastic food contact materials, which has passed the enforcement tests of compliance, unknown migrants out of the legislation lists, such as EU No 10/2011, may still exist. Based on the above, in the present study, a GC-MS scan method was used to screen the migrants in a panel of 120 “qualified” plastic FCM. Interestingly, a number of unlisted migrants were discovered, whereas an additional quantification and analysis was performed toward identifying the high-risk migrants.

## Materials and methods

### Chemicals and reagents

The reference materials, Bis(2-ethylhexyl) adipate (DEHA), Bis(2-ethylhexyl) phthalate (DEHP), stearamide and 2,4-di-tert-butylphenol (2,4-DTBP), were purchased from Dr. Ehrenstorfer Company (Augsburg, Germany). The other chemical reagents were from E. Merck Company (Darmstadt, Germany). All chemicals and reagents used in GC-MS were of chromatographic grade without further purification.

#### Samples

Plastic FCM products were collected from the Chinese national product supervisory inspection from 2016 to 2017. The samples covered all authorized categories of plastic FCM products in China, including bottles, disposable plastic tableware, plastic drink bottles, plastic wrap bags, packing bags, etc. The materials were classified as poly(ethylene terephthalate) (PET), polyethylene (PE), polycarbonate (PC), polypropylene (PP), low-density polyethylene plastics (LDPE), melamine-formaldehyde (MF), polystyrene (PS) and biaxially oriented polypropylene/low density polyethylene(BOPP/LDPE).

#### Qualification tests of plastic FCM products

The enforced qualification tests of plastic FCM products were conducted according to the Chinese national standard GB 4806.7–2016[25]. Firstly, the overall migration was evaluated using hexane as food stimulant. The pre-treatment methods for migration test of specimens (including immersion rules, calculation of sample area) are operated according to GB5009.156[26]. Then, two hundred millilitres of extract were transferred into evaporating dish in water bath until all liquids were evaporated. The dish was put into oven in 100°C±5°C for 2 hours to remove all the solvent. In the end cooled the dish in the desicator for 30 min. And then weight increase was estimated. The overall migration was calculated and expressed as weight increase per-square decimeter.

Subsequently, the specific migrations were determined according to the different FCM materials. The material types, target migrants, related SML and determination methods were shown in Table 1. After the qualification experiments, the products with an overall migration lower than 10 mg/dm^2^ and a specific migration lower than SML were considered as qualified products for further analysis.

**Table 1:** The material types and the specific migration compounds.

Materials	Target compounds	Methods	Limitations (SML)
PET	Acetaldehyde,	GC	15 μg/g (QM)^a^
	Antimony	ICP	0.04 mg/kg(SML)
PE	-	-	-
PC	BPA	LC-MS	0.6 mg/kg(SML)
PP	-	-	-
MF	Melamine	LC	2.5 mg/kg(SML)
	Formaldehyde	Spectrophotometry	215 mg/kg(SML(T))^b^
PS	Butadiene	GC	ND(DL = 0.01 mg/kg, SML)^c^
	Ethylbenzene	GC	0.3%(QM)^d^;
	Styrene	GC	0.5%(QM)
BOPP/LDPE	Diaminotoluene	GC	0.004 mg/kg(SML)
	Solvent residue	GC	5.0 mg/m^2^(QM)
	Benzene residue	GC	ND(DL = 0.01 mg/m^2^,QM)

^a^ The limitation is fit for colorless PET bottle, mechanical crushing.

^b^ SML(T)- total specific migration limit means the maximum permitted sum of particular substances released in food or food stimulants expressed as total of moiety of the substances indicated.

^c^ ND- not detected; DL- detection limit.

^d^ QM- maximum residue limit.

### Screening of chemical migrants in FCM products

According to the result of migrants in enforcement tests, we choose 120 random samples to thoroughly investigate the migrants in these materials, we use 10 mL hexane to resolve all the migrants in the glass dish, and then the extract was filtered through a 0.22 μm syringe filter. A GS-MS method was employed to scan possible chemical migrants in the “qualified” specimen. The GC-MS analysis was carried out on an Agilent 7890B Ultra GC system equipped with an electron impact (EI) source operating at 70eV in full scan mode. The separation was performed on Agilent HP-5MS analytical column (30m×0.25mm, 0.25μm) using 99.999% high purity helium as carrier gas at a flow rate of 1 mL per min. The injection volume was 1 μL without split, the injector temperature was 250°C and the detector temperature was 280°C. The oven temperature was programmed from 60°C to 220°C at 20°C·min^-1^ and hold at this temperature for 1min, and finally rise to 280°C (at 5°C·min^-1^) and kept for 4 min.

Migration compounds in the FCM products were identified from the full scan data (from m/z 35 to 500). Peak discrimination and integration was carried out using the MSD Chemstation F.01.03.2357 software (2012 version, Agilent Technologies, Inc.). Subsequently, the chemical structures were determined based on the NIST/EPA/NIH Mass Spectral Library (2011version, Agilent Technologies, Inc.).

### Analysis of overall migrant profile

All detected compounds were categorized according to their chemical structure. To describe the overall profile of all migrants, further analyses were performed. In all types of materials, the amount of occurrences for different substances was calculated, and the compounds contained in the list of European Regulation EU No 10/2011 were also indicated. Then, among all detected migrants, chemical substances considered hazardous and thus been adopted on the Norwegian Priority List[6] of hazardous substances or the REACH Candidate list of SVHC-substances were selected for further quantitative analysis. Meanwhile, several other substances that had been indicated in previous studies[20] as potential risk factors were also selected for the quantification test.

### Quantification of migrants with potential risk

#### DEHA and DEHP

The detection procedure was according to the method described in the literature (DEHA[27], DEHP[28]). The FCM plastic was rinsed with HPLC-grade water and crushed into pieces. Then 0.2 g of samples were accurately weighted and extracted with 10 mL of n-hexane in ultrasonic bath for 3 consecutive times. After mixing the extracts, the solution was concentrated into 1mL in pure N_2_ and filtered through 0.22 μm syringe filters into a microvial before injection into the GC-MS detector system. DEHA and DEHP analyses were carried out using GC-MS (Agilent 7890B) in the selected ion monitoring (SIM) mode. The extract was injected into an HP-5MS analytical column (30 m×0.25 mm, 0.25 μm) in splitless mode. Helium was used as carrier gas, and hydrogen/air was used for the flame. The injector and detector temperature was 250°C and 280°C, respectively. The temperature program was as follows: 60°C for 1min; increasing to 220°C at 20°C·min^-1^ and holding for 1min; increasing to 280°C at 5°C·min^-1^ and holding for 4min.

#### Stearamide and 2,4-DTBP

The detection procedure was according to the method described in the literature (Stearamide[29], 2,4-DTBP [30]). After rinsing with HPLC-grade water, 2 g of the samples were accurately weighted and dissolved in 50 mL ethyl acetate in ultrasonic bath, and then filtered and evaporated at 90 ^o^C with rotary. The residue was dissolved in 2 mL pure methanol and then analyzed by high performance liquid chromatography (HPLC) with DAD detector. An Agilent 1200 series HPLC (Palo Alto, CA, USA), consisting of an online degasser, a quaternary pump, a thermostated column compartment, auto-sampler and a diode-array detector, was used. The Agilent Technologies’ LC1260 Open Lab ChemStation software was employed for data analysis. The column used in stearamide analysis was a reverse phase C18 column (150×4.6 mm i.d.) from Agilent Company (USA). DAD detector operated at 202 nm. Mobile phase was a mixture of acetonitrile: methanol (60:40, V/V) at 0.5 mL·min^-1^ flow rate whereas the injection volume of the sample was 20μL. Separation was carried out at 25±3°C. The analysis of 2,4-DTBP was performed in the same column, but its mobile phase was a mixture of water: methanol: isopropanol(25:60:15, V/V) at 1.0 mL·min^-1^ flow rate and operated at 270 nm.

## Results

### Overall chemical migrants in plastic FCM

**Fig 1:**
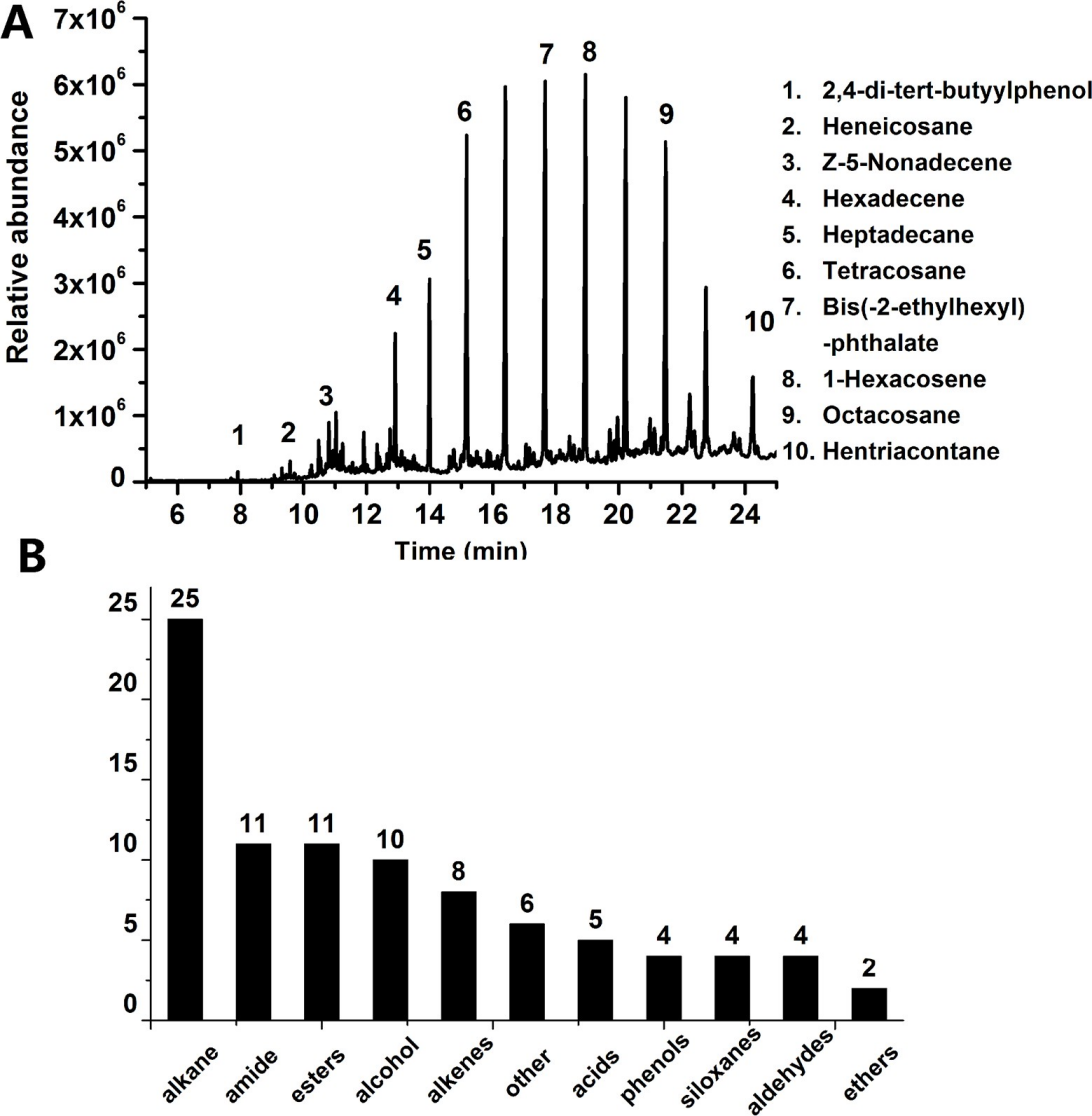
(A) Indicative chromatograph of chemical migrants in a BOPP/LDPE film. (B) Identified chemical types in the 120 qualified FCM samples. The compounds were determined by GC-MS scan method and the standard spectrum library of NIST.

After the enforced qualification tests, one hundred twenty qualified plastic FCM products were obtained for the analysis of unknown chemical migrants. Chemical migrants were screened on a GC-MS platform and identified by the NIST/EPA/NIH Mass Spectral Library. On the basis of the obtained results, dozens or hundreds of different components were appeared in the chromatograph of each sample, and some chemicals were identified. The presentative chromatogram of the migrants in a BOPP/LDPE film sample was shown in Fig 1A. In the hexane extract of this specimen, more than 15 compounds were appeared and 10 of them were identified. The Total Ion Chromatography (TIC) curves were used to determine the relative amount of the compounds from the GC-MS analysis. The migrants detected with the GC-MS scan in all samples were listed in S1 Table.

### Migrants profile in different FCM materials

According to their chemical structure, all migrants were classified into 11 categories: alkanes, amides, esters, alcohols, alkenes, acids, phenols, siloxanes, aldehydes, ethers and other substances. The different chemical compounds in each category were shown in Fig 1B.

Then, identified migrants in the different materials were analyzed. Table 2 summarizes the main chemical categories of migrants in relation to the seven investigated types of materials. The alkane and phenolic substances were found almost in all FCM products. In PC products, the amide chemicals identified were only oleamide and erucamide, without stearamide. In PET products, the amount of chemical substances was significantly lower than that in other materials (P < 0.05), and no amides were detected. Squalene was only found in the PP material. MF materials contained only alkanes and esters (Bis(2-ethylhexyl), phthalate and Dioctyl terephthalate).

**Table 2:** Existence of identified chemicals in FCM material types.

Materials	alkane	amide	esters	alcohol	alkenes	acids	phenols	siloxanes	aldehydes	ethers	others
PET(n = 23)	23	0	2	0	5	0	5	10	0	0	0
PE(n = 30)	30	30	22	2	8	8	22	2	2	0	2
PC(n = 5)	5	3	4	0	0	1	4	0	0	0	0
PP(n = 35)	27	20	9	3	5	15	24	0	8	5	0
MF(n = 9)	9	0	4	0	0	0	0	0	0	0	0
BOPP/LDPE(n = 11)	5	10	6	5	2	2	7	3	0	0	4
PS(n = 7)	2	0	0	0	0	0	5	0	0	0	0

In addition, the potential impact of the different identified migrants to human health was assessed. The toxicology data of all chemicals were retrieved, and most substances were of low toxicity. However, despite of their origin, only 12 chemicals in all identified migrants were existed in the EU No 10/2011 list of the permitted chemicals for FCM plastic manufacture (Table 3). Further, among all migrants, three components were involved in the Norwegian Priority List (Erik Hansen, 2013) of hazardous substances, REACH Candidate List of SVHC-substances[31] or potential risk factors mentioned in previous literature: DEHA, DEHP, and stearamide. Although 2,4-DTBP was not involved in any priority list, we found that it exists in all types of FCM materials. To determine the accurate content of these chemicals in FCM products and accumulate data for future risk assessment, quantitative analysis was subsequently performed.

**Table 3:** The EU No 10/2011 permitted additives/starting substance in identified chemicals.

No	CAS No.	Compound	Molecular	Mr	Detection rate
1	112-41-4	1-Dodecene	C12H24	168.32	3%
2	301-02-0	Oleamide	C18H35NO	281.48	64%
3	124-26-5	Stearamide	C18H37NO	283.49	32%
4	112-84-5	cis-13-Docosenoamide	C22H43NO	337.58	59%
5	64-18-6	Formic acid	CH2O2	46.03	2%
6	88-99-3	Pathalic acid	C8H6O4	166.13	3%
7	57-10-3	Palmitic acid	C16H32O2	256.42	26%
8	112-92-5	1-Hydroxyoctadecane	C18H38O	270.49	2%
9	661-19-8	1-Docosanol	C22H46O	326.6	5%
10	103-23-1	Bis(2-ethylhexyl) adipate	C22H42O4	370.57	12%
11	117-81-7	Bis(2-ethylhexyl) phthalate	C24H38O4	390.56	52%
12	6422-86-2	Dioctyl terephthalate	C24H38O4	390.56	12%

### Quantitative analysis of potential risk factors

The quantitative determination of four chemical substances (DEHP, DEHA, stearamide and 2,4-DTBP) was performed using GC-MS or HPLC method. Table 4 depicts the chemical characteristics, the quantitative results and the corresponding limits of the above mentioned substances. As shown in Table 4, the amount of DEHP and DEHA in different type of FCM materials were lower than the SML in Commission Regulation EU No 10/2011, whereas that of stearamide was under the recommended limit (60 mg/kg). The 2,4-DTBP was widely existed in the seven types of FCM products except MF. Among them, the highest level, up to 45.568±31.513 mg/kg, was obtained in BOPP/LDPE materials.

**Table 4:** The quantitative results of 4 potential risk factors in FCM samples.

	DEHA	DEHP^a^	2,4-DTBP	Stearamide
**LOD**^b^ **(mg/kg)**	0.005	0.005	0.010	10.000
**Method Recovery (%)**	97	104	95	107
**PP(QM) (mg/kg)**	3.322±0.594	0.294±0.027	2.431±0.815	14.070±1.640
**PET(QM) (mg/kg)**	ND	ND	1.335±0.347	ND
**BOPP/LDPE(QM) (mg/kg)**	3.509±1.015	0.244±0.017	45.568±31.513	39.763±17.392
**PE(QM) (mg/kg)**	3.779±0.207	0.171±0.001	25.557±22.211	20.573±13.265
**PC(QM) (mg/kg)**	ND	0.243±0.007	1.260±0.385	ND
PS(QM)(mg/kg)	ND	ND	3.907±1.672	ND
**MF(SML) (mg/kg)**	ND	0.009±0.004	ND	ND

^a^ According to directive EU No 10/2011, the SML of DEHA and DEHP is 18mg/kg and 1.5mg/kg, respectively.

^b^ LOD- limit of detection.

## Discussion

Most FCM plastics and related products are generally considered as “inert” materials when they used as container or package, even under heating or microwave. Actually, the authoritative frameworks of safety guarantee for FCMs, as well as the testing and evaluation methods have been well-established. To date, almost all domestic and export FCM products are imposed to strict compliance tests before sale. Furthermore, new knowledge on polymer chemistry and chemical toxicology is continuously introduced to improve the standards and detection technologies, thus ameliorating the whole level of safety for FCM plastics[32].

On the other hand, it is possible that some chemicals without authoritative evaluation could be added by some manufacturers. This might bring new potential risks if undetected chemicals migrate from FCM into food. In this study, we have presented an effective GC-MS approach to monitor unknown chemical migrants in a series of “qualified” plastic FCM products. Theoretically, these products are safe for use because of their low overall and specific migration. Unexpectedly, thousands of chemical substances were found, from which nearly 100 were identified. It seems that plastic FCMs were not so “inert”, especially when contacting to greasy food.

In particular, only 13% of the identified chemicals were included in the permitted starting materials of EU No 10/2011. In other words, most of the identified migrants were not imposed to safety evaluation tests. A basic analysis involving the chemical structure, the material type, the toxicology data and the applications of migrants was proposed in order to thoroughly describe their profile. The identified chemicals were originated from different sources (contaminants or functional agents) depending on materials type and FCM production procedure. For instance, mineral oil (long chain alkane) and phenols might migrate from the ink, while chlorinated paraffin, Bis(2-ethylhexyl) adipate, Bis(2-ethylhexyl) and phthalate dioctyl terephthalate were used as plasticizing agents during the polymerization process[33]. Pathalic acid was the precursor of polymerization process whereas cis-13-Docosenoamide and octadecanol were used as lubricant additives for the production of various products[34].

Numerous safety evaluations of these compounds have been under investigation. We have chosen four most representative risk factors for further quantitative determination. Both of the DEHA and DEHP are plasticizers, a former research has found that a significant increase in stomach tumors was observed in the females rats due to the exposure of DEHA[35]. Borch et al [36] showed that perinatal DEHP exposure in male rats reduced steroidogenesis. Two recent studies revealed that long-term exposure or repeated contact of DEHP may contribute to the development of liver adenomas and carcinomas[37, 38]. As an example of phenolic antioxidant, 2,4 DTBP have been widely used as an intermediate for the preparation of antioxidants, UV stabilizers and in manufacturing of pharmaceuticals and fragrances[39, 40]. Stearamide, a widely used smooth agent, is considered as potential risk factor by OW Lauet al [19]. Although there is no obviously direct evidence that the quantitative results of these 4 potential risk factors in plastic have toxic to the expected human consumption and exposure, the complex contents of the plastic is still worth our attention. There are still plenty of works to study and investigate deeply.

## Conclusions

A GC-MS scan method was employed to monitor the various chemical substances/migrants in qualified FCM products. For several chemicals that were identified as potential risk factors, a quantification analysis was in addition carried out. It’s the first study that massively explore the chemical migrants in a wide group of plastic FCMs. Unexpectedly, numerous substances were detected, whereas only the 13% was included in the permitted list of directive EU No 10/2011. Several known highly risky compounds, such as DEHP, DEHA, 2,4-DTBP and stearamide, were below the recommended limits. In conclusion, the plastic FCMs were not so “inert” as they usually considered. Further evaluation studies toward the limitation of new identified compounds should be performed.

## Supporting information

S1 Table The migrants discovered with the GC-MS scan in all samples. (DOCX)
