# Supplementary material for: Detection and quantification analysis of chemical migrants in plastic food contact products
Source: PLoS One. 2018 Dec 5;13(12):e0208467. doi: 10.1371/journal.pone.0208467 (PMC6281260; doi:10.1371/journal.pone.0208467)
Supplement: S1 Table — (DOCX) [file pone.0208467.s001.docx]

**S1 Table.** **The migrants discovered with the GC-MS scan in all samples.**

| **No.** | **Types** | **Compound** | **Molecular** | | **Mr** | | | | **CAS No.** | | | | | | |
| --- | --- | --- | --- | --- | --- | --- | --- | --- | --- | --- | --- | --- | --- | --- | --- |
| 1 | alkanes | n-Hendecane | C_11_H_24_ | | 156.31 | | | | 1120-21-4 | | | | | | |
| 2 | alkanes | Cyclododecane | C_12_H_24_ | | 168.32 | | | | 294-62-2 | | | | | | |
| 3 | alkanes | 2-Methyl-hexadecane | C_17_H_36_ | | 240.47 | | | | 1560-92-5 | | | | | | |
| 4 | alkanes | n-Heptadecane | C_17_H_36_ | | 240.47 | | | | 629-78-7 | | | | | | |
| 5 | alkanes | 3-Methylheptadecane | C_18_H_38_ | | 254.49 | | | | 6418-44-6 | | | | | | |
| 6 | alkanes | n-Eicosane | C_20_H_42_ | | 282.55 | | | | 112-95-8 | | | | | | |
| 7 | alkanes | Phytane | C_20_H_42_ | | 282.55 | | | | 638-36-8 | | | | | | |
| 8 | alkanes | Nonadecane, 9-methyl- | C_20_H_42_ | | 282.55 | | | | 13287-24-6 | | | | | | |
| 9 | alkanes | n-Heneicosane | C_21_H_44_ | | 296.57 | | | | 629-94-7 | | | | | | |
| 10 | alkanes | n-Tetracosane | C_24_H_50_ | | 338.65 | | | | 646-31-1 | | | | | | |
| 11 | alkanes | n-pentacosane | C_25_H_52_ | | 352.68 | | | | 629-99-2 | | | | | | |
| 12 | alkanes | n-hexacosane | C_26_H_54_ | | 366.71 | | | | 630-01-3 | | | | | | |
| 13 | alkanes | Heneicosane, 11-(1-ethylpropyl)- | C_26_H_54_ | | 366.71 | | | | 55282-11-6 | | | | | | |
| 14 | alkanes | n-HEPTACOSANE | C_27_H_56_ | | 380.73 | | | | 593-49-7 | | | | | | |
| 15 | alkanes | n-Octacosane | C_28_H_58_ | | 394.76 | | | | 630-02-4 | | | | | | |
| 16 | alkanes | n-Nonacosane | C_29_H_60_ | | 408.79 | | | | 630-03-5 | | | | | | |
| 17 | alkanes | [n-TRIACONTANE](javascript:showMsgDetail('ProductSynonyms.aspx?CBNumber=CB3162103&postData3=CN&SYMBOL_Type=A');) | C_30_H_62_ | | 422.81 | | | | 638-68-6 | | | | | | |
| 18 | alkanes | n-Hentriacontane | C_31_H_64_ | | 436.84 | | | | 630-04-6 | | | | | | |
| 19 | alkanes | n-Hentriacontane | C_31_H_64_ | | 436.84 | | | | 630-04-6 | | | | | | |
| 20 | alkanes | 11-Decyldocosane | C_32_H_66_ | | 450.88 | | | | 55401-55-3 | | | | | | |
| 21 | alkanes | 11-Decyltetracosane | C_34_H_70_ | | 478.934 | | | | 55429-84-0 | | | | | | |
| 22 | alkanes | n-Pentatriacontane | C_35_H_72_ | | 492.95 | | | | 630-07-9 | | | | | | |
| 23 | alkanes | Pentacosane, 13-undecyl- | C_36_H_74_ | | 506.988 | | | | 55517-89-0 | | | | | | |
| 24 | alkanes | n-Tetratetracontane | C_44_H_90_ | | 619.19 | | | | 7098-22-8 | | | | | | |
| 25 | alkanes | Alkane C54 | C_54_H_110_ | | 759.45 | | | | 5856-66-6 | | | | | | |
| 26 | alkenes | 1-Dodecene | C_12_H_24_ | | 168.32 | | | | 112-41-4 | | | | | | |
| 27 | alkenes | 2-Cyclohexyloctane | C_14_H_28_ | | 196.37 | | | | 2883-05-8 | | | | | | |
| 28 | alkenes | 1-Nonadecene | C_19_H_38_ | | 266.51 | | | | 18435-45-5 | | | | | | |
| 29 | alkenes | 1-Docosanol | C_22_H_44_ | | 308.59 | | | | 1599-67-3 | | | | | | |
| 30 | alkenes | 9-Hexacosene | C_26_H_52_ | | 364.69 | | | | 71502-22-2 | | | | | | |
| 31 | alkenes | 1-Hexacosene | C_26_H_52_ | | 364.702 | | | | 18835-33-1 | | | | | | |
| 32 | alkenes | 9-Vinylanthracene | C_16_H_12_ | | 204.27 | | | | 2444-68-0 | | | | | | |
| 33 | alkenes | Squalene | C_30_H_50_ | | 410.72 | | | | 111-02-4 | | | | | | |
| 34 | amides | Hexadecanamide | C_16_H_33_NO | | 255.44 | | | | 629-54-9 | | | | | | |
| 35 | amides | 9-Octadecenamide | C_18_H_35_NO | | 281.484 | | | | 4303-70-2 | | | | | | |
| 36 | amides | Oleamide | C_18_H_35_NO | | 281.48 | | | | 301-02-0 | | | | | | |
| 37 | amides | Octadecanamide | C_18_H_37_NO | | 283.49 | | | | 124-26-5 | | | | | | |
| 38 | amides | cis-13-Docosenoamide | C_22_H_43_NO | | 337.58 | | | | 112-84-5 | | | | | | |
| 39 | amides | N-Tetradecanamide | C_14_H_29_NO | | | 227.39 | | | | 638-58-4 | | | |  |  |
| 40 | amides | N-[pyridin-3-yl-[2-(trifluoromethyl)phenyl]imino-methyl]hydroxylamine | C_13_H_10_F_3_N_3_O | | | 281.08 | | | | 288246-53-7 | | | |  |  |
| 41 | amides | Pentafluoropropionic acid tetradecyl ester | C_17_H_29_F_5_O_2_ | | | 360.4 | | | | 6222-06-6 | | | |  |  |
| 42 | amides | Heptafluorobutyric acid tetradecyl ester | C_18_H_29_F_7_O_2_ | | | 410.41 | | | | 7365-36-8 | | | |  |  |
| 43 | amides | 1-Chloroicosane | C_20_H_41_Cl | | | 316.99 | | | | 42217-02-7 | | | |  |  |
| 44 | amides | 1-Chloroheptacosane | C_27_H_55_Cl | | | 415.187 | | | | 62016-79-9 | | | |  |  |
| 45 | siloxanes | Decamethylcyclopentasiloxane | C_10_H_30_O_5_Si_5_ | 370.77 | | | | 541-02-6 | | | |  |  |  |  |
| 46 | siloxanes | Dodecamethylcyclohexasiloxane | C_12_H_36_O_6_Si_6_ | | | | 444.92 | | | | 540-97-6 | | | |  |
| 47 | siloxanes | 15H-Hexadecamethyloctasiloxane | C_16_H_48_O_7_Si_8_ | | | | 577.233 | | | | 19095-24-0 | | | |  |
| 48 | siloxanes | [Octadecamethylcyclononasiloxane](http://www.baidu.com/link?url=ChGV0ZGwqripZADy12cXYROnm5IeVj2ysPw0NvrG5tCmXmg9IkORf82OtDugUMXSA9n-Yq-U8s7llC_jpxqSRVCcHOWTbWGv0S3EYw1_OpKBk9rfSscQmanXXGiYMr1k) | C_18_H_54_O_9_Si_9_ | | | | 667.39 | | | | 556-71-8 | | | |  |
| 49 | acids | Formic acid | CH_2_O_2_ | | | | 46.03 | | | | 64-18-6 | | | |  |
| 50 | acids | Sodium carbonate | CNa_2_O_3_ | | | | 105.99 | | | | 497-19-8 | | | |  |
| 51 | acids | Pathalic acid | C_8_H_6_O_4_ | | | | 166.13 | | | | 88-99-3 | | | |  |
| 52 | acids | Palmitic acid | C_16_H_32_O_2_ | | 256.42 | | | | 57-10-3 | | | |  |  |  |
| 53 | acids | Nonahexacontanoic acid | C_69_H_138_O_2_ | | 999.861 | | | | 40710-32-5 | | | |  |  |  |
| 54 | alcohols | 1-Octyn-3-ol | C_8_H_14_O | | 126.2 | | | | 818-72-4 | | | |  |  |  |
| 55 | alcohols | 5-Indanol | C_9_H_10_O | | 134.18 | | | | 1470-94-6 | | | |  |  |  |
| 56 | alcohols | 2-Hexyl-1-decanol | C_16_H_34_O | | 242.44 | | | | 2425-77-6 | | | |  |  |  |
| 57 | alcohols | 1-Heptadecanol | C_17_H_36_O | | 256.47 | | | | 1454-85-9 | | | |  |  |  |
| 58 | alcohols | 1-Hydroxyoctadecane | C_18_H_38_O | | 270.49 | | | | 112-92-5 | | | |  |  |  |
| 59 | alcohols | 1-Nonadecanol | C_19_H_40_O | | 284.52 | | | | 1454-84-8 | | | |  |  |  |
| 60 | alcohols | 1-Docosanol | C_22_H_46_O | | 326.6 | | | | 661-19-8 | | | |  |  |  |
| 61 | alcohols | 2-Monopalmitin | C_19_H_38_O_4_ | | 330.5 | | | | 23470-00-0 | | | |  |  |  |
| 62 | alcohols | 1-Tetracosanol | C_24_H_50_O | | 354.65 | | | | 506-51-4 | | | |  |  |  |
| 63 | alcohols | 1,2-Dipalmitoyl-sn-glycerol | C_35_H_68_O_5_ | | 568.91 | | | | 761-35-3 | | | |  |  |  |
| 64 | aldehydes | 3,5-Dimethylbenzaldehyde | C_9_H_10_O | | 134.18 | | | | 5779-95-3 | | | |  |  |  |
| 65 | aldehydes | 4-N-propylbenzaldehyde | C_10_H_12_O | | 148.2 | | | | 28785-06-0 | | | |  |  |  |
| 66 | aldehydes | 4,4,5,7-Tetramethyl-2-chromanone | C_13_H_16_O_2_ | | 204.267 | | | | 40662-14-4 | | | |  |  |  |
| 67 | aldehydes | 3,5-Di-tert-butyl-4-hydroxybenzaldehyde | C_15_H_22_O_2_ | | 234.334 | | | | 1620-98-0 | | | |  |  |  |
| 68 | esters | Tridecyl acrylate | C_16_H_30_O_2_ | | 254.41 | | | | 3076-04-8 | | | |  |  |  |
| 69 | esters | 7,9-Ditert-butyl-1-oxaspiro[4.5]deca-6,9-diene-2,8-dione | C_17_H_24_O_3_ | | 276.376 | | | | 82304-66-3 | | | |  |  |  |
| 70 | esters | Phthalic acid, 6-ethyloct-3-yl methyl ester | C_19_H_28_O_4_ | | 320.429 | | | |  | | | |  |  |  |
| 71 | esters | Butyl 2-ethylhexyl phthalate | C_20_H_30_O_4_ | | 334.45 | | | | 85-69-8 | | | |  |  |  |
| 72 | esters | 2-Monostearin | C_21_H_42_O_4_ | | 358.56 | | | | 621-61-4 | | | |  |  |  |
| 73 | esters | Bis(2-ethylhexyl) adipate | C_22_H_42_O_4_ | | 370.57 | | | | 103-23-1 | | | |  |  |  |
| 74 | esters | Phthalic acid, di(2-propylpentyl) ester | C_24_H_38_O_4_ | | 390.55 | | | | 70910-37-1 | | | |  |  |  |
| 75 | esters | Diiscooctyl phthalate | C_24_H_38_O_4_ | | 390.564 | | | | 27554-26-3 | | | |  |  |  |
| 76 | esters | Bis(2-ethylhexyl) phthalate | C_24_H_38_O_4_ | | 390.56 | | | | 117-81-7 | | | |  |  |  |
| 77 | esters | Dioctyl terephthalate | C_24_H_38_O_4_ | | 390.56 | | | | 6422-86-2 | | | |  |  |  |
| 78 | esters | Diisooctyl Phthalate | C_24_H_38_O_4_ | | 390.564 | | | | 131-20-4 | | | |  |  |  |
| 79 | phenols | 2,5-Bis(1,1-dimethylethyl)-pheno | C_14_H_22_O | | 206.32 | | | | 5875-45-6 | | | |  |  |  |
| 80 | phenols | 2,4-Di-tert-butylphenol | C_14_H_22_O | | 206.32 | | | | 96-76-4 | | | |  |  |  |
| 81 | phenols | 4-tert-Octylphenol | C_14_H_22_O | | 206.32 | | | | 140-66-9 | | | |  |  |  |
| 82 | phenols | 4-tert-Butyl-2,6-diisopropylphenol | C_16_H_26_O | | 234.38 | | | | 57354-65-1 | | | |  |  |  |
| 83 | ethers | ARALDITE M | C_13_H_18_O_2_ | | 206.283 | | | | 3101-60-8 | | | |  |  |  |
| 84 | ethers | di-N-dodecyl ether | C_24_H_50_O | | 354.65 | | | | 4542-57-8 | | | |  |  |  |
| 85 | other | 2,6-Di-tert-butyl-p-benzoquinone | C_14_H_20_O_2_ | | 220.31 | | | | 719-22-2 | | | |  |  |  |
| 86 | other | tridecyloxirane | C_15_H_30_O | | 226.398 | | | | 18633-25-5 | | | |  |  |  |
| 87 | other | 3-Acetylphenanthrene | C_16_H_12_O | | 220.27 | | | | 2039-76-1 | | | |  |  |  |
| 88 | other | Oleanitrile | C_18_H_33_N | | 263.461 | | | | 112-91-4 | | | |  |  |  |
| 89 | other | 2-Methylindole | C_9_H_9_N | | 131.17 | | | | 95-20-5 | | | |  |  |  |
| 90 | other | n-Heptadecanonitrile | C_17_H_33_N | | 251.45 | | | | 5399-02-0 | | | |  |  |  |
